# Brain Health and Traumatic Brain Injury (TBI): Relationship Between Brain Care Score and Disability in Subacute-Long Term TBI

**DOI:** 10.1177/15598276261472823

**Published:** 2026-08-08

**Authors:** Maral Sakayan, Maria T. Paulino, Amanda S. Fang, Claire Joyner, Stephania Tovar-Vargas, Arunima Kapoor, Alexis Conrad, Michael Lopez, Saef Izzy, Scott M. Bartell, Danh V. Nguyen, Mark Mapstone, Bernadette Boden-Albala, Patrick M. Chen

**Affiliations:** 1 Neurology Traumatic Brain Injury & Concussion (NTBIC) Program, Department of Neurology, 8788 University of California, Irvine, Orange, CA, USA (MS, MTP, ASF, CJ, ST-V, AK, AC, ML, PMC); 2 Department of Neurology, School of Medicine, 8788 University of California Irvine, Irvine, CA, USA (MS, MM, BB-A, PMC); 3 Department of Health Society and Behavior, Joe C Wen School of Population and Public Health, 8788 University of California Irvine, Irvine, CA, USA (ST-V, SB, BB-A); 4 Department of Epidemiology and Biostatistics, Joe C Wen School of Population and Public Health, University of California Irvine, Irvine, CA, USA (ST-V, SB, BB-A); 5 Department of Neurology, Harvard Medical School, 1861 Brigham and Women’s Hospital, Boston, MA, USA (SI); 6 Department of Environmental and Occupational Health, Joe C Wen School of Population and Public Health, 8788 University of California Irvine, Irvine, CA, USA (SB); 7 Department of Medicine, 8788 University of California Irvine, Irvine, CA, USA (DVN)

**Keywords:** traumatic brain injury, brain care score, brain health, psychosocial, disability, exposome

## Abstract

Traumatic brain injury (TBI) is a prevalent, chronic disease with heterogenous recovery and dementia risk. Poor brain health is associated with increased dementia risk, but brain health remains unexplored in TBI. Brain Care Score (BCS), a tool measuring brain health with physical, lifestyle, and social factors, has not been used in TBI. Retrospective cohort study of TBI-clinic visits in the Neurology Traumatic Brain Injury and Concussion Clinic (9/2022-10/2025). Inclusion criteria: ≥18yo, self-reported TBI, BCS, functional outcomes. Disability defined as Glasgow Outcome Scale-Extended (GOSE) <7. Among 185 patients (median age 45yo, 55.1% men, 76.2% mild TBI), 64.9% were disabled (mean GOSE 5.11 [SD: 0.80]) whom had lower BCS vs non-disabled (14.9 vs 16.8, *P* < 0.001). In multivariable analysis adjusted for age, sex, insurance status, Injury Severity Score (ISS), higher ISS was associated with greater odds of disability (OR = 1.06, 95% CI: 1.02-1.10, *P* = 0.006). Higher BCS was associated with lower odds of disability (OR = 0.779, 95% CI: 0.677-0.883, *P* < 0.001). We demonstrate feasibility of measuring brain health with BCS in a TBI clinic. Higher BCS is associated with lower disability risk. Future studies will investigate brain health as potentially intervenable risk factors for long-term disability in TBI survivors.

“The majority of our cohort had low-medium brain health, highlighting the TBI population as a vulnerable population for future brain health-related diseases like dementia.”

## Introduction

Traumatic brain injury (TBI) is a prevalent and chronic disease^1,2^ with heterogenous recovery trajectories and dementia risk. Nearly 10 million Americans, or 3.3% of all American adults, in 2023, sustained a TBI within that past year.^3^ Although this high incidence of TBI exists, there is a lack of concrete data to predict functional disability in post-TBI at the subacute or chronic stage of their disease.^4^ It is known that specific brain-related factors like history of prior TBI and premorbid psychiatric conditions are risk factors of prolonged TBI functional recovery, yet the role of brain health—defined by the American Academy of Neurology (AAN) as “a continuous state of attaining and maintaining optimal neurologic function”^5^—on TBI functional outcomes remain underexplored.

A recent joint statement between the AAN and the American Heart Association (AHA) emphasizes that brain health is “crucial to optimizing both the function and well-being of every person at each stage of life and is key to both individual and social progress.”^5^ Conversely, poor brain health is associated with increased risks of stroke,^6,7^ dementia,^6,7^ late-life depression,^8^ cardiovascular disease,^9^ specific cancers (lung, colorectal, and breast),^9^ white matter hyperintensities,^10^ and cerebrovascular events (in middle-aged women).^11,12^

Though there exists no gold-standard measure of brain health to date, the Brain Care Score (BCS), established by the McCance Center for Brain Health from Massachusetts General Hospital,^6^ created through a modified Delphi process, is a potential tool. BCS examines an individual’s Physical, Lifestyle, and Social Emotional factors and provides a value on a scale of 0-21 with higher BCS indicating better brain health.^6^ A large analysis of the UK Biobank database showed the general trend that a higher BCS was associated with a lower incidence of several health conditions. Specifically, a 5-point higher BCS was associated with a 59% lower risk of dementia in those <50 years old (Hazard Ratio (HR): 0.41, 95% Confidence Interval (CI): 0.28-0.60), while a 5-point higher BCS was associated with 52% lower risk of stroke among in those 50-59 years old (HR: 0.48, 95% CI: 0.44-0.53).^7^ This lends support to the idea of yet-to-be determined BCS components (such as blood pressure, alcohol use, social relationships, etc.) potentially serving as modifiable risk factors in preventing these conditions.

Overall, brain health is a potential modifiable risk factor for specific chronic conditions, including dementia and stroke. However, the role of brain health and the evaluation of brain health through tools like BCS is unexplored in long-term TBI outcomes. We hypothesize that poor brain health, as measured by a lower BCS score, is associated with increased disability in subacute to chronic TBI. Here we aim to examine the cross-sectional association between BCS and concurrent self-reported disability in a post-TBI clinic cohort and explore the feasibility of using the BCS in a TBI clinic cohort.

## Methods

### Design

This was a retrospective cohort study of a prospective collection of patients with TBIs at the University of California (UCI) Neurology Traumatic Brain Injury and Concussion (NTBIC) Clinic.

### Standard Protocol Approvals, Registrations, and Patient Consents

We obtained the data from an ongoing institutional review board (IRB) approved clinical data registry at the University of California, Irvine (IRB # 2283). The requirement for informed consent was waived.

### Participants

Study participants are from the UCI NTBIC database, which consists of all post-TBI patients seen in the TBI outpatient clinic from September 2022 to October 2025. The NTBIC clinic is an academic post-TBI clinic at a Level 1 Trauma Center in Orange County, CA, and evaluates TBI patients discharged from the UCI emergency/trauma departments and from community referral for TBI with persistent symptoms. Inclusion criteria consisted of patients 18 years or older, awake and communicative, with a reported suspected or definite TBI history per the 2023 American Congress of Rehabilitation Medicine (ACRM) criteria.^13^ Glasgow Coma Scale (GCS) at admission at first provider report per chart review and both radiographic positive (intraparenchymal, intradural, epidural or subarachnoid blood, skull fracture) and negative (no aforementioned hemorrhage in the brain nor skull fracture) TBI are included. Primary language of English and Spanish were included. At TBI outpatient clinic visits, specifically at index visits, BCS forms (available in both English and Spanish) were given to each patient to complete. If patients were communicative but unable to fill out the BCS forms due to physical injury, their proxy was permitted to complete the form. If patients were severely cognitively impaired or unable to communicate, they were excluded. Mechanism of injury was corroborated by clinician via chart review. Exclusion criteria included inability to corroborate TBI and mechanism of injury with outside documentation, premorbid disability, ongoing medical-legal litigation, and incomplete data.

### Brain Care Score

The BCS, first introduced from the McCance Center for Brain Health from Massachusetts General Hospital, is a tool to encourage lifestyle changes to lower one’s risk of dementia, stroke, and late-life depression.^6–8^ It was later extrapolated to estimate risk in cardiovascular disease and cancer.^9^ This 21-point tool is divided into three large categories: Physical, Lifestyle, and Social Emotional, which is further sub-categorized. Scores are given based on responses to Criteria and Descriptions of sub-categories, with a maximum total of 21 points.^6^ BCS quintiles determined by previous large studies which used the following quintiles: 3-13 (1st quintile), 14-15 (2nd quintile), 16-17 (3rd quintile), 18 (4th quintile), and 19-21 (5th quintile).^6^ The quintiles were then grouped as low BCS (1st quintile), medium BCS (2nd–4th quintiles), and high BCS (5th quintile).^6^ These quintiles were utilized in this study given the similarity in outcomes investigated with previous methods.

#### Measures

Demographic information including age, sex, race, ethnicity, and insurance status is collected as standard of care in all NTBIC patients via standardized clinical interview. Characteristics of TBI injury including mechanism of injury, time from TBI to clinic visit, Injury Severity Score (ISS), severity of TBI (based on GCS), as well as premorbid medical conditions (cardiovascular disease includes hypertension, coronary artery disease, hyperlipidemia, etc., and psychiatric disease includes anxiety, depression, etc.) are collected via standardized interview and corroborated with chart review by a trained extractor. TBI severity was measured by ACRM criteria for mild TBI and by Department of Veterans Affairs, Department of Defense (VA DOD) for moderate to severe TBI, and validated via chart review.

#### Functional Outcomes

##### Primary Outcome

Functional disability status was measured using the Glasgow Outcome Scale-Extended (GOSE). The definition of disabled was less than a score of 7 on GOSE.

The GOSE is a widely used metric of TBI disability and scored on a scale from 1 to 8 with 1 indicating death, 2 indicating a vegetative state, 3 to 6 reflecting moderate-severe disability, and 7 to 8 considered good recovery. Dichotomized outcome (1-6 vs 7-8) is the same as previous large multi-center TBI studies.^14^ Outcome measures are self-reported by standard written questionnaire at a patient’s index clinical visit and standardized patient interview per past studies^15^ as needed, representing disability at first visit.

#### Statistical Analysis

Descriptive statistics and comparison analysis were performed to compare disabled vs non-disabled participants and to compare low, medium, and high BCS participants. We inspected histograms and box-whisker plots to determine non-normality. We therefore used non-parametric testing, specifically Mann-Whitney-U test for continuous variables or Chi-square/Fisher-Freeman-Halton test for categorical variables accordingly. The primary outcome, GOSE, was modeled as a dichotomized categorical variable in multivariable logistic regression. The primary exposure of interest was BCS. Multivariable logistic regression was adjusted for age, sex, insurance status, ISS, informed by group consensus and previous studies to suggest ISS as an important variable in understanding polytrauma and TBI role in disability.^16^ Odds Ratio greater than 1 (OR > 1) indicated greater odds of disability and OR <1 indicates lower odds of disability (i.e., protective association). GOSE was dichotomized for the primary analysis, consistent with larger TBI studies and reflecting a clinically realistic patient-centric threshold for recovery.^14,17,18^ A sensitivity analysis was conducted using ordinal logistic regression for further granularity in disability. Additional sensitivity analysis compared disabled vs non-disabled mild TBI patient cohorts. To evaluate the relative impact of specific brain health components, we categorized individual BCS factors into three broad domains (Endogenous, Behavioral, and Social) and performed a multivariable logistic regression analysis adjusting for age, sex, and ISS. Spearman’s rank correlation coefficient was performed to assess the strength and direction of a monotonic relationship between GOSE and BCS. Statistical significance was defined as *P* < 0.05. Statistical analysis was performed with R Studio v. 4.3.2 (Boston, MA).

#### Data Availability

The data that support the findings of this study are available on request from the corresponding author. Due to privacy or ethical restrictions, the data are not publicly available.

#### Reporting Guideline

We used the STROBE reporting guideline^19^ to draft this manuscript, and the STROBE reporting checklist^19^ when editing (supplement A).

## Results

### Patient Characteristics

This study consisted of 185 TBI patients (after removal of 12 patients who did not complete BCS forms for unknown reasons), of whom 141 had mild TBI, with most patients seen in our outpatient clinic by 5 months of their head injury [interquartile range (IQR): 2-24]. The median age of this cohort was 45 years [IQR: 31-61] with 85 patients (45.9%) in the “midlife” age group (40-65 years old) and 77 patients (41.6%) in the “young” age group (<40 years old). Over half of patients were male (55.1%). Half (51.4%) of patients were White, 2.7% were Black, 21.8% were Asian, and the remaining 24.9% were of Other race. Nearly a quarter of this cohort (28.6%) was either uninsured or enrolled in Medicaid. The mean BCS was 15.6 [standard deviation (SD): 3.10] and the mean GOSE score was 5.93 [SD: 1.32] for the entire study population.

### Differences Between Disabled and Non-disabled Subjects

There were 120 patients in the disabled group with GOSE <7 (mean GOSE 5.11 [SD: 0.80], while there were 65 patients in the non-disabled group defined as GOSE 7-8 (mean GOSE 7.45 [SD: 0.50]). There were no significant differences in age, sex, race, time from TBI to clinic visit, mechanism of TBI, positive findings on head imaging, severity of TBI, and premorbid conditions (i.e., cardiovascular and psychiatric conditions); see Table 1. Patients in the disabled group tended to have no insurance or Medicaid (35.0% vs 16.9%, *P* = 0.015). Disabled TBI patients had lower BCS compared to non-disabled (14.9 [SD: 2.95] vs 16.8 [SD: 3.05], *P* < 0.001). When comparing the BCS sub-categories, there were no significant differences in BMI, diet, alcohol consumption, and smoking between disabled vs non-disabled groups; see Table 1. Patients in the disabled group were less likely to engage in aerobic activities (39.2% vs 75.4%, *P* < 0.001), routinely have less than 7 hours of sleep (58.0% vs 41.3%, *P* = 0.046), report moderate to high levels of stress (58.8% vs 34.9%, *P* = 0.004), lack social interaction (26.9% vs 6.3%, *P* = 0.002), and struggle with meaning in life (32.8% vs 9.5%, *P* = 0.001). Overall, examination of individual BCS components shows differences in lifestyle and social-emotional factors, while physical domain variables (BMI, diet, alcohol, smoking) did not significantly differ between groups.

**Table 1.** Differences Among Disabled and Non-disabled Subjects.

​	Non-disabled (GOSE ≥ 7) n = 65	Disabled (GOSE < 7) n = 120	*P*-value
Age (median [IQR])	39 [23.00-63.00]	46 [34.00-58.00]	0.199
“Midlife” (40-65 years old)	21 (24.7%)	64 (75.3%)	​
“Young” (<40 years old)	35 (45.5%)	42 (54.5%)	​
Sex female (n, %)	29 (44.6%)	54 (45.0%)	1.000
Race (n, %)			0.115
White	30 (46.2%)	65 (54.2%)	​
Black	1 (1.5%)	4 (3.3%)	​
Asian	20 (30.8%)	19 (15.8%)	​
Other	14 (21.5%)	32 (26.7%)	​
Non-White race (n, %)	35 (53.8%)	55 (45.8%)	0.375
Non-Hispanic ethnicity (n, %)	54 (84.4%)	88 (73.3%)	0.130
Medicaid or No insurance (n, %)	11 (16.9%)	42 (35.0%)	0.015
Time from TBI to clinic visit (months; median [IQR])	5.00 [2.00-25.00]	5.00 [2.00-23.25]	0.578
Premorbid medical conditions			
Cardiovascular conditions (n, %)	13 (20.0%)	28 (23.3%)	0.737
Psychiatric conditions (n, %)	12 (18.5%)	36 (30.0%)	0.125
Severity of TBI, based on GCS (n, %)			0.073
Mild TBI	55 (84.6%)	86 (71.7%)	​
Moderate-severe TBI	10 (15.4%)	34 (28.3%)	​
Injury severity score (median [IQR])	2.00 [1.00-9.00]	5.00 [1.00-17.00]	0.058
Brain care score (mean [SD])	16.8 [3.05]	14.9 [2.95]	<0.001
Premorbid hypertension (n, %)	11 (16.9%)	19 (15.8%)	1.000
Premorbid diabetes or HLD (n, %)	9 (13.8%)	23 (19.2%)	0.478
BMI (mean [SD])	25.52 [4.10]	25.91 [5.36]	0.611
Issues with healthy diet (n, %)	11 (17.5%)	26 (21.8%)	0.613
Alcohol consumption (n, %)	14 (21.5%)	14 (11.7%)	0.116
Smoking (n, %)	6 (9.2%)	12 (10.0%)	1.000
Aerobic activities (n, %)	49 (75.4%)	47 (39.2%)	<0.001
Routine sleep of 7-8 hours (n, %)	26 (41.3%)	69 (58.0%)	0.046
Moderate-high stress levels (n, %)	22 (34.9%)	70 (58.8%)	0.004
Lack of social interaction (n, %)	4 (6.3%)	32 (26.9%)	0.002
Lack of meaning in life (n, %)	6 (9.5%)	39 (32.8%)	0.001

### Multivariable Analysis

In multivariable analysis adjusting for age, sex, insurance status, ISS, a higher ISS was independently associated with greater odds of disability (OR [Odds ratio]: 1.06, 95% CI: 1.02-1.10, *P* = 0.006). Conversely, a higher BCS demonstrated a significant inverse association (protective) with disability (OR: 0.779, 95% CI: 0.677-0.883, *P* < 0.001); see Table 2.

**Table 2.** Multivariable Logistic Regression Assessing the Association Between BCS and Disability. The Model is Adjusted for Age, Sex, Insurance Status, and Injury Severity Score (ISS).

​	Odds Ratio (95% CI)	*P*-value
Age	1.01 (0.994-1.03)	0.187
Sex female (ref: Male)	1.55 (0.778-3.16)	0.218
Medicaid/No insurance (ref: Private insurance)	2.08 (0.909-4.99)	0.090
Injury severity score	1.06 (1.02-1.10)	0.006
Brain care score	0.779 (0.677-0.883)	<0.001

### Spearman’s Rank Correlation

**Figure 1.**
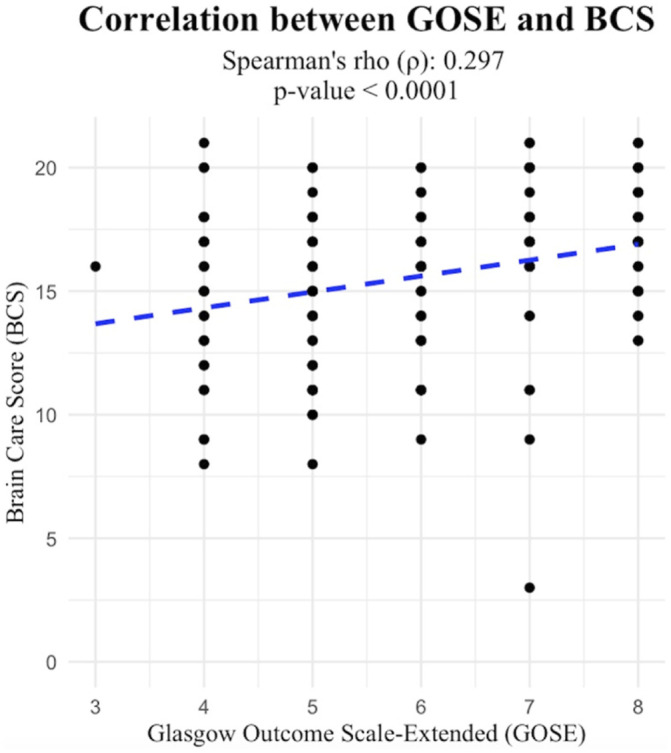
Correlation between GOSE and BCS. The Spearman’s rank correlation coefficient (ρ) was 0.297, with a significant low positive correlation between GOSE and BCS (*P* < 0.0001).

Spearman’s rho of GOSE and BCS was 0.297 (*P* < 0.001), consistent with a significant low positive correlation between GOSE and BCS (Figure 1).

### Stratified Analysis by BCS Group and Age

There were 42 patients in the low BCS group (1st quintile), 114 patients in the medium BCS group (2nd–4th quintiles), and 29 patients in the high BCS group (5th quintile). There were no significant differences in sex, race, time from TBI to clinic visit, cumulative number of hits to the head, mechanism of TBI, and premorbid conditions among these groups. Those with lower BCS scores were older, with the low BCS category having the oldest age compared to the medium BCS and high BCS groups (51 years [IQR: 40.50-58.75]) vs 44 [IQR: 33-62 vs 31 [IQR: 23-56], *P* = 0.034). A higher portion of low and medium BCS reported previous TBI (31.0% and 39.5%) compared to high BCS (10.3%, *P* = 0.011). In an age-stratified correlation analysis, the association between BCS and functional outcome was more pronounced and statistically significant in the midlife cohort (rho = 0.265, *P* = 0.014) compared to the younger cohort (rho = 0.201, *P* = 0.080).

### Sensitivity Analyses

An ordinal logistic regression was performed as a sensitivity analysis and GOSE was represented as an ordinal scale (GOSE 3 “severe disability” – GOSE 8 “upper good recovery”). Both ISS (OR: 0.925, 95% CI: 0.900-0.953, *P* < 0.001) and BCS (OR: 1.23, 95% CI: 1.12-1.36, *P* < 0.001) remained significantly associated with functional outcome; see Table 3. No patients in this cohort had GOSE scores of 1 or 2.

**Table 3.** Sensitivity Analysis Using Ordinal Logistic Regression for Association Between BCS and Disability. The Model Accounts for Age, Sex, Insurance Status, and Injury Severity Score.

​	Odds Ratio (95% CI)	*P*-value
Age	0.989 (0.973-1.00)	0.170
Sex female (ref: Male)	0.730 (0.429-1.24)	0.245
Medicaid/No insurance (ref: Private insurance)	0.876 (0.468-1.64)	0.678
Injury severity score	0.925 (0.900-0.953)	<0.001
Brain care score	1.23 (1.12-1.36)	<0.001

In a comparison of only mild TBI patients (n = 141), there were no significant differences between the disabled and non-disabled groups regarding age, sex, race, or ISS (Table 4). However, patients in the non-disabled group demonstrated significantly higher mean BCS compared to those with disability (16.76 vs 14.60; *P* < 0.001).

**Table 4.** Differences Between Disabled and Non-disabled Mild TBI Patients.

​	Non-disabled (GOSE ≥ 7) n = 55	Disabled (GOSE < 7) n = 86	*P*-value
Age (median [IQR])	39.00 [23.00-62.50]	51.00 [36.25-60.75]	0.152
Female (n, %)	29 (52.7%)	45 (52.3%)	1.000
Race (n, %)			0.664
White	27 (49.1%)	51 (59.3%)	​
Black	1 (1.8%)	2 (2.3%)	​
Asian	14 (25.5%)	17 (19.8%)	​
Other	13 (23.6%)	16 (18.6%)	​
Non-White race (n, %)	28 (50.9%)	35 (40.7%)	0.310
Non-Hispanic ethnicity (n, %)	44 (81.5%)	69 (80.2%)	1.000
Medicaid or No insurance (n, %)	9 (16.4%)	26 (30.2%)	0.097
Time from TBI to clinic visit (months; median [IQR])	4.00 [2.00-8.00]	4.00 [2.00-19.75]	0.725
Premorbid medical conditions			
Cardiovascular conditions (n, %)	11 (20.0%)	22 (25.6%)	0.576
Psychiatric conditions (n, %)	12 (21.8%)	28 (32.6%)	0.235
Injury severity score (median [IQR])	1.00 [1.00-6.00]	1.00 [1.00-8.25]	0.933
Brain care score (mean [SD])	16.76 [3.14]	14.60 [2.79]	<0.001

To further investigate the components of the BCS, we performed an exploratory multivariable analysis assessing the three individual BCS domains (Endogenous, Behavioral, and Social) in relation to disability. When adjusting for age, sex, and ISS, the Social Domain demonstrated a robust and independent association with disability (OR: 2.59; 95% CI: 1.74-4.05; *P* < 0.001). In contrast, the Endogenous (OR: 1.04; *P* = 0.859) and Behavioral (OR: 1.11; *P* = 0.591) domains were not significantly associated with disability in this model. These findings suggest that the social components of the BCS may be the most significant modifiable drivers of functional status in this clinical TBI population (Supplemental Table 1).

## Discussion

This retrospective cohort study of a prospective collection of TBI patients examines brain health in mild to severe TBI seen in an outpatient clinic during their recovery course. We demonstrate the feasibility of assessing brain health in TBI using the BCS, an instrument developed to optimize brain health by directing patient and clinician focus onto potentially intervenable risk factors in the development of age-related brain disorders. The BCS serves as a tool to obtain objective metrics related to brain health. We found that higher BCS, along with lower ISS, was concurrently associated with more favorable functional outcomes following TBI at clinic presentation. In other words, we show that a patient’s BCS and ISS at their index clinic visit was associated with their functional recovery at the time of that clinic visit, adjusting for classic TBI variables. While the BCS has been validated in its association with stroke and dementia, the tool was created to serve as a proxy for overall brain health, lending itself to broader application across brain-related diseases including TBI, where the tool has not previously been applied.

We highlight the BCS as a publicly available, low administration burden, easy-to-use (from our experience, 5 minutes or less to complete) screening tool in a post-TBI outpatient setting. Similar conclusions have been drawn in other non-TBI studies, showing the utilization and adaptability of the BCS in a variety of clinical settings.^8^ This study highlights the feasibility of measuring brain health in a brain-injury clinic setting, and is a platform for future study of how modifiable brain health factors may influence functional recovery following TBI. A strength of this study is that while prior research utilizing the BCS relies on retrospective methods, our study collected BCS in a “real world” clinic, prospectively.

It is important to highlight that the majority of our cohort is mild TBI with a high rate of disability. This speaks to the growing consensus that “mild” TBI has a previously underappreciated rate of chronic symptoms and societal disability.^20^ Those with mild TBI are still vulnerable to self-reported disability post-TBI. This further supports that TBI is a chronic medical condition, and additional studies and investigation about post-TBI outcomes are critical.

Individual components of the BCS, such as hypertension,^21^ hyperglycemia, obesity,^17^ smoking,^18^ poor sleep quality,^22,23^ stress,^24,25^ and social isolation^26,27^ have already been implicated as modulators of neuronal injury and long-term outcomes throughout animal and clinical models of TBI. In this study, we implement the BCS in TBI with the goal of integrating these existing, discrete findings via an accessible clinical tool. In TBI, the BCS synthesizes cardiovascular, metabolic, and psychosocial variables, providing a snapshot of the cumulative environment in which the injured brain is actively recovering, and possibly, the synergistic effects among these variables. Several aspects of the BCS align with the emerging research in the role of neuroinflammation in TBI^24,26^ as well as in the importance of social support^26–28^ during the recovery course. For example, chronic stress, isolation, and hyperglycemia can lead to excess glucocorticoids that induce microglial priming and an increase in inflammatory cytokines leading to chronic inflammation.^24^ Similarly, cardioembolic syndromes, likely driven by adipose tissue dysfunction, may exacerbate inflammatory cascades and increase symptom burden post-TBI.^17^ Our findings suggest Lifestyle and Social-Emotional domains within BCS are the primary drivers of disability in this TBI population. While disabled patients frequently reported a lack of aerobic exercise and poor sleep, the absence of social interaction and a sense of meaning in life was particularly striking. These findings suggest that social life factors may carry an underappreciated disproportionate weight in TBI recovery compared to traditional metabolic markers. However, because lifestyle and social variables are interrelated, this study serves as a first step toward a domain-based approach, highlighting a critical gap in our understanding of how to effectively measure and treat these social-behavioral factors. Future research utilizing an emerging “exposome” (the totality of life-exposures experienced) informed framework is necessary to understand these complex relationships and develop targeted, personalized interventions within a comprehensive brain health model.

The majority of our cohort had low-medium brain health, highlighting the TBI population as a vulnerable population for future brain health-related diseases like dementia. Patients in the lowest BCS quintile in this cohort were a median 20 years older than those in the highest quintile. On further sub-analysis of age groups, there is a correlation with lower BCS and midlife-elderly age; this may suggest the aging brain is correlated with lower brain health, and thus a vulnerable and targetable population. These findings may reflect a cumulative burden on brain reserve, via age or TBI-related mechanisms. Given the increasing awareness toward modifiable risk factors for dementia, which includes a history of TBI,^29,30^ broader implementation of the BCS provides a tangible, widely accessible, clinical tool for monitoring and discussing two disease processes that are already mechanistically linked. Higher BCS has been associated with decreased risk of dementia and stroke across all age groups along with a lower incidence of late-life depression.^8^ Further, a higher BCS is associated with lower incidence of cardiovascular disease and three common cancer types (lung, breast and colorectal).^9^ The BCS may be one future method to objectively risk stratify for future neurologic and non-neurologic disease. While no consensus exists on what constitutes a clinically meaningful BCS—nor defined thresholds for “good” or “bad”—our study adds to growing evidence that higher BCS values are associated with better clinical outcomes than lower values.^9–12^ Overall, the BCS is a dynamic value. Future studies can measure brain health with BCS to address potentially intervenable risk factors and to evaluate if early intervention of these can reduce chronic disease and disability.

It is important to note that there is no current gold standard of brain health measurement. The self-reported metric nature of BCS allows for reduction in provider bias given the utilization of a scale to quantify brain health. Other proposed measures of brain health include the Brain Health Index (BHI),^30^ created to aid in risk stratification for cognitive impairment, and The LIfestyle for BRAin health risk score (LIBRA),^31^ designed to estimate dementia risk. The BHI includes risk factors for cognitive impairment and modifiable lifestyle factors contributing to cognitive resilience. The BHI, though multi-dimensional, utilizes normalized and weighted subsections, making it more time-intensive for participants. The LIBRA consists of 12 lifestyle factors, each weighted based on their protective or negative contribution to dementia risk. Notably, current brain health tools are largely weighted toward cardiometabolic health. This is relevant given emerging studies suggest poor cardiometabolic health and obesity worsen TBI outcomes due to chronic inflammation.^17,32,33^ Future studies will need to compare BCS to BHI and LIBRA, in addition to better understanding brain health-related factors specific to TBI.

Limitations exist in this study. Outcome measures such as GOSE, although commonplace in large scale TBI studies, fail to capture nuanced aspects of patient recovery and are prone to recall bias. While our definition of optimal BCS as the highest quintile is consistent with that of the largest BCS study, to the best of our knowledge, there is no formal cutoff or dichotomization for BCS to date. Additionally, dichotomization of any variable inherently reduces granularity. The self-reported nature of BCS can also introduce bias, or be considered as confounding factors, as patients may intentionally or unintentionally report inaccurate lifestyle factors. However, investigating lifestyle factors is inherently challenging. Furthermore, BCS is a composite measure that reflects multiple domains of patient health and lifestyle. These domains may themselves be shaped by underlying health and socioeconomic factors, which could influence disability but were not fully accounted for by the covariates included in our model. Finally, the exclusion of patients with incomplete BCS data and use of proxy-data, may introduce selection and information bias (i.e., severe cognitive impairment may have been less likely to complete the assessment). As such, residual confounding factors may contribute to the observed association between BCS and functional outcome. The results of this study reflect a TBI population that seeks care at a California Academic Level 1 Trauma Center with a specialized TBI clinic and may not generalize to community settings. BCS forms were available in English and Spanish only, thus excluding those who speak other languages. Further, our inclusion criteria required patients to be awake and communicative to fill out the BCS forms (or have their proxies do so). This inherently excludes patients who are non-communicative and possibly with increased rates of functional disability. Although we utilize sub-analyses for age and TBI severity, we acknowledge the overall heterogeneity of this TBI population, limiting external validity. Lastly, this analysis was performed in a cross-sectional associative framework; therefore, claims regarding temporality and causation cannot be made. This study cannot determine if BCS represents a general marker of poor health of those susceptible to TBI. In other words, this study cannot determine directionality of this relationship between BCS and post-TBI outcomes, nor prediction of disability directly. Future longitudinal studies will investigate if targeted modification of specific BCS domains—through shared clinician-patient decision making—is associated with prospective changes in functional outcome.

## Conclusion

Though we cannot draw conclusions of causation, we found higher BCS values have an association with lower rates of self-reported disability in post-TBI patients. Ultimately, this study highlights that brain health is an understudied but important metric in a vulnerable TBI patient population, especially in the aging brain. We highlight the feasibility of gathering BCS data in a real-life TBI clinic cohort. Future studies will investigate how targeting changes in specific BCS sub-categories can potentially modify an individual’s BCS, overall brain health, and influence disability.

## Supplemental Material


Supplemental Material - Brain Health and Traumatic Brain Injury (TBI): Relationship Between Brain Care Score and Disability in Subacute-Long Term TBI
Supplemental Material for Brain Health and Traumatic Brain Injury (TBI): Relationship Between Brain Care Score and Disability in Subacute-Long Term TBI by Maral Sakayan, Maria T. Paulino, Amanda S. Fang, Claire Joyner, Stephania Tovar-Vargas, Arunima Kapoor, Alexis Conrad, Michael Lopez, Saef Izzy, Scott Bartell, Danh V. Nguyen, Mark Mapstone, Bernadette Boden-Albala and Patrick M. Chen in American Journal of Lifestyle Medicine.

Supplemental Material - Brain Health and Traumatic Brain Injury (TBI): Relationship Between Brain Care Score and Disability in Subacute-Long Term TBI
Supplemental Material for Brain Health and Traumatic Brain Injury (TBI): Relationship Between Brain Care Score and Disability in Subacute-Long Term TBI by Maral Sakayan, Maria T. Paulino, Amanda S. Fang, Claire Joyner, Stephania Tovar-Vargas, Arunima Kapoor, Alexis Conrad, Michael Lopez, Saef Izzy, Scott Bartell, Danh V. Nguyen, Mark Mapstone, Bernadette Boden-Albala and Patrick M. Chen in American Journal of Lifestyle Medicine.
