# Supplementary material for: Brain Health and Traumatic Brain Injury (TBI): Relationship Between Brain Care Score and Disability in Subacute-Long Term TBI
Source: Am J Lifestyle Med. 2026 Aug 8:15598276261472823. Online ahead of print. doi: 10.1177/15598276261472823 (PMC13452889; doi:10.1177/15598276261472823)
Supplement: Supplementary file 2 [file sj-pdf-2-ajl-10.1177_15598276261472823.pdf]

Supplemental Table 1. Multivariable logistic regression three BCS domain associations with disability. The model accounts for age, sex, and injury severity score.

	Odds Ratio (95% CI)	p-value
**Age**	1.014 (0.993-1.036)	0.188
**Sex Female** (ref: Male)	1.365 (0.665-2.851)	0.401
**Injury Severity Score**	1.071 (1.030-1.120)	<0.001
**Endogenous Domain**	1.041 (0.670-1.633)	0.859
**Behavioral Domain**	1.113 (0.755-1.651)	0.591
**Social Domain**	2.594 (1.744-4.054)	<0.001
